# Supplementary material for: Identification of photoperiod-induced specific miRNAs in the adrenal glands of Sunite sheep (Ovis aries)
Source: Front Vet Sci. 2022 Jul 22;9:888207. doi: 10.3389/fvets.2022.888207 (PMC9354845; doi:10.3389/fvets.2022.888207)
Supplement: Supplementary file 1 [file Data_Sheet_1.ZIP › Supplementary file/miRDeep2 file.zip!/miRDeep2 file/Know_Structure/LP42A2/oar-mir-299.pdf]

miRBase precursor : oar-mir-299
Total read count : 216
oar-miR-299-5p read count : 212
oar-miR-299-3p read count : 4
remaining reads : 0

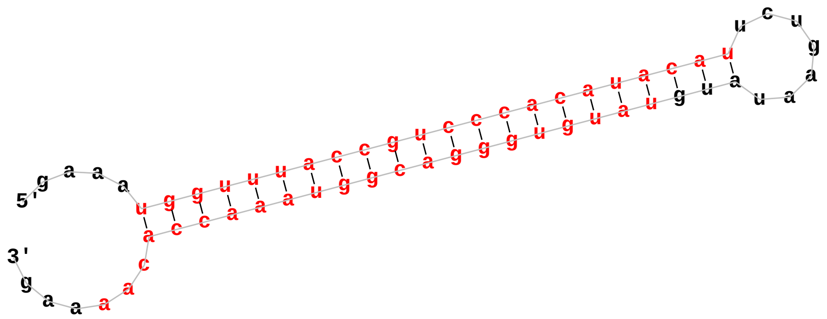

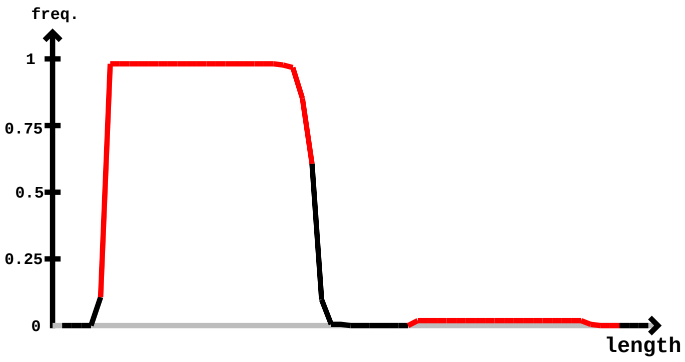


```
         oar-miR-299-5p
                                      oar-miR-299-3p
5'- gaaaugguuuaccgucccacauacauucugaauauguaugugggacgguaaaccacaaaag   -3'    exp
    ....((((((((((((((((((((((.......))))))))))))))))))))))......    reads      mm         sample
    ...augguuuaccgucccacaua......................................    1          0          seq
    ...augguuuaccgucccacauac.....................................    7          0          seq
    ...augguuuaccgucccacauaca....................................    15         0          seq
    ....ugguuuaccgucccacau.......................................    1          0          seq
    ....ugguuuaccgucccacaua......................................    1          0          seq
    ....ugguuuaccgucccacauac.....................................    18         0          seq
    ....ugguuuaccgucccacauaca....................................    38         0          seq
    ....ugguuuaccgucccacauacau...................................    110        0          seq
    ....ugguuuaccgucccacauacauA..................................    7          1          seq
    ....ugguuuaccgucccacauacauu..................................    13         0          seq
    ....ugguuuaccgucccacauacauucu................................    1          0          seq
    ....................................uaugugggacgguaaacc.......    3          0          seq
    ....................................uaugugggacgguaaacca......    1          0          seq
```
